# On choosing the vehicles of metaphors 2.0: the interactive effects of semantic neighborhood density and body-object interaction on metaphor production

**DOI:** 10.3389/fpsyg.2023.1216561

**Published:** 2023-09-05

**Authors:** Hamad Al-Azary, Albert N. Katz

**Affiliations:** ^1^Department of Humanities, Social Sciences and Communication, Lawrence Technological University, Southfield, MI, United States; ^2^Department of Psychology, The University of Western Ontario, London, ON, Canada

**Keywords:** metaphor, embodied cognition, semantic richness, body object interaction, semantic neighborhood density

## Abstract

In a metaphor, such as *language is a bridge*, two distinct concepts known as the topic (i.e., *language*) and vehicle (i.e., *bridge*) are juxtaposed to produce figurative meaning. Previous work demonstrated that, when creating metaphors, participants choose vehicles that are concrete, rather than abstract, and are also a moderate semantic distance away from the topic. However, little is known about the semantic representations underlying metaphor production beyond topic-vehicle semantic distance and vehicle concreteness. Here, we studied the role of two semantic richness variables in metaphor production – semantic neighborhood density (SND), which measures the proximity of a word and its associations in semantic space, and body-object interaction (BOI), which reflects the ease with which a human body can motorically interact with a word’s referent. In each trial, participants were presented with an abstract topic, such as *miracle*, and were instructed to make an apt and comprehensible metaphor by choosing a vehicle word (e.g., *lighthouse*). All of the topics were abstract but half were high-SND (from dense semantic neighborhoods) and half were low-SND (from sparse semantic neighborhoods). Similarly, half of the potential vehicle words were either high or low in SND and also differed on BOI such that half were high-BOI (e.g., *bicycle*), whereas half were low-BOI (e.g., *rainbow*). We observed a three-way interaction such that participants selected low-BOI, rather than high-BOI, vehicle words when topics or vehicles were high-SND. We interpret this finding to suggest that participants attempt to reduce the overall semantic richness of their created metaphors.

## Introduction

The majority of psycholinguistic studies on metaphor focus on comprehension, leaving the important question of metaphor production poorly understood (Holyoak and Stamenković, 2018). Moreover, those few studies that examine metaphor production tend to focus on a variety of questions, such as pragmatic factors that promote metaphor production (Hussey & Katz, 2006); brain networks involved in metaphor production (Beaty et al., 2017); the role of working memory in metaphor production (Chiappe and Chiappe, 2007); individual differences in semantic networks in creative metaphors (Li et al., 2021); or the distinction between metaphor and simile production (Oka et al., 2022). While these studies characterize multiple facets of metaphor production, they do not address a more basic elementary question; namely, what kinds of concepts are chosen to produce good metaphors? That is, why is it apt to describe one’s lawyer as a *shark*, and perhaps less apt (though conventional) to describe a faulty car as a *lemon*? In this paper, we characterize the semantic representations that make for producing comprehensible novel metaphors.

Katz (1989) employed a vehicle selection paradigm to bring metaphor production under experimental control. In his task he asked participants to complete metaphor frames (e.g., *Sociology is the ________ of sciences*) by choosing the single best word from a list to serve as a vehicle (e.g., *robin*, *hawk*, *USA*, *Switzerland*, etc.). Two primary semantic variables of the selected vehicles were analyzed, word concreteness and semantic distance to the topic. Both concreteness and semantic distance effects were observed. Participants chose concrete vehicles more often than they did abstract vehicles, and the vehicles that were chosen were a moderate distance from the topic, rather than near or far. Therefore, Katz showed that a vehicle’s concreteness and its position among other concepts in semantic space both play a role in producing metaphors.

In this paper, we extend on the work of Katz (1989) by characterizing the semantic representations of topics and vehicles in metaphor production beyond semantic distance and concreteness. Again, a vehicle selection task was employed wherein participants were provided with abstract words that served as metaphor topics, and provided with a word-bank of concrete words to serve as vehicles. We focused on two semantic variables that are related to the variables studied by Katz (1989).

First, while Katz examined the semantic distance between topic and vehicle, we considered the semantic neighborhood density (SND) of the topic and vehicle words, which characterizes the proximity of a word and its neighbors in semantic space (Buchanan et al., 2001). Second, although Katz demonstrated that concrete words are preferred to use as metaphor vehicles, it remains unclear what aspects of concreteness are important. One aspect of concrete concepts is their motoric affordances (Gibson, 1979). Some concepts afford motoric interaction, such as *bicycle*, in as much as we interact with the referent motorically in many ways, whereas with others, such as *butterfly* we have less motoric interaction. Therefore, it is unclear if motoric interaction is the driver of concreteness effects in metaphor production. To investigate this, we considered body-object interaction (BOI), which characterizes the ease with which one can interact with a word’s referent (Siakaluk et al., 2008). Below, we describe these variables in greater detail and how they may pertain to metaphor processing.

### Body-object interaction

Body-object interaction has been studied in a number of lexical and semantic decision studies (Siakaluk et al., 2008), with the common finding that high-BOI words such as *bicycle* are responded to faster than low-BOI words such as *butterfly*. Concepts that are high in BOI are argued to be semantically richer, such that they contain more semantic information, which facilitates their relatively rapid activation. However, BOI effects are task dependent; Tousignant and Pexman (2012) reported that BOI behavioral effects are cancelled out when they are expected (see also Muraki et al., 2023). From an embodied cognition perspective, BOI concepts may be processed faster because they are more amenable for sensorimotor simulations (Siakaluk et al., 2008). This is consistent with neuroimaging findings indicating that high-BOI concepts activate kinesthetic brain regions (Hargreaves et al., 2012). Moreover, in tasks emphasizing motoric interaction (i.e., *does the word refer to something that is touchable*?), high-BOI and low-BOI concepts evoke distinct early electrophysiological effects (Al-Azary et al., 2022). The present study is the first to examine the role of BOI in metaphor processing, whereby participants created metaphors by choosing vehicles that, although are all concrete, vary on BOI. Focusing on BOI allows one to tease apart the aspects of concreteness that drive metaphor production; if high-BOI concepts are favored in metaphor production, it would suggest that motoric properties of vehicles are a primary source of metaphorical meaning. Conversely, if low-BOI concepts are favored in metaphor production, it would suggest that other modalities related to concreteness, such as visual imagery, play a primary role in constructing metaphorical meaning.

### Semantic neighborhood density

In addition, to body-object interaction, we also considered semantic neighborhood density (SND), which is a measure of the average similarity between a word and its nearest associations (Buchanan et al., 2001). Semantic neighborhood density has been demonstrated to affect metaphor comprehension in a variety of online and offline processing tasks (Al-Azary and Buchanan, 2017; Al-Azary et al., 2019, 2021). In sum, the aforementioned studies on SND and metaphor comprehension demonstrate that words from sparser, rather than denser, semantic neighborhoods, result in more comprehensible metaphors. Al-Azary and colleagues argued that semantically dense words have little “room” to take-on novel metaphorical associations. Here, we examine if SND affects metaphor production in the same manner as metaphor comprehension. We focused on the vehicle’s SND, in order to determine if this variable interacts with BOI. Moreover, we also considered the SND of the topic, as greater topic SND has been shown to negatively affect metaphor interpretations (Reid et al., 2020).

In the present study, participants were presented with abstract words to serve as metaphor topics (e.g., *secrecy*) and selected a vehicle word from a word-bank (e.g., *rat*) to create novel metaphors (i.e., *secrecy is a rat*). We opted to restrict the topics to abstract concepts because abstract concepts are typically grounded metaphorically with concrete concepts (e.g., Lakoff and Johnson, 2003; Barsalou, 2012). The topic words varied on semantic neighborhood density and the vehicle words varied on both semantic neighborhood density in addition to body-object interaction.

## Method

### Participants

Sixty people participated, each for a compensation of $10 CAD. Participants were recruited from poster advertisements around campus, and the summer participant research pool at the University of Western Ontario. Participants reported being native speakers of English.

### Materials

#### Topics

Abstract words, determined by their ratings in the Brysbaert et al. (2014) concreteness norms, were used as topics. Words were chosen from the lowest concreteness quartile and therefore are the most abstract. Semantic neighborhood density was derived from the WINDSORS database (Durda and Buchanan, 2008), a global co-occurrence model used in previous metaphor processing research (Al-Azary and Buchanan, 2017; Al-Azary et al., 2021), and was defined as the average semantic distance between a word and its neighbors within 3.5 standard deviations away. A median split value was used to categorize words as either high or low-SND. Eighteen of the topics are high SND whereas 18 are low SND. Furthermore, topics were low-frequency, ranging from 1–40 per million words, also defined in the WINDSORS database. The abstract-high SND and abstract-low SND topics did not differ significantly on abstractness, *t* (34) = 1.0076, *p* = 0.32, or frequency, *t* (34) = 0.34, *p* = 0.74 but as expected, differed on SND, *t* (34) = 9.24, *p* < 001. See Table 1 for the full list of topics.

**Table 1:** Words serving as topics and their respective semantic conditions.

High SND	Low SND
Eternity	Luck
Euphoria	Legacy
Courage	Revenge
Loyalty	Irony
Repentance	Prestige
Honesty	Destiny
Epiphany	Imagination
Serenity	Betrayal
Empathy	Nostalgia
Patience	Temptation
Obsession	Innocence
Ambition	Persuasion
Sadness	Metaphor
Narcissism	Miracle
Guilt	Boredom
Sincerity	Secrecy
Hatred	Solitude
cowardice	Amusement

#### Potential vehicles

The items chosen as potential vehicles are concrete nouns (all rated greater than 4.5 on a 5-point concreteness scale; Brysbaert et al., 2014) but differed on BOI. Body-object interaction is determined from normed studies; here, we used databases of multisyllabic (Bennett et al., 2011) and monosyllabic (Tillotson et al., 2008) nouns. To develop a suitable list of items, as a starting point, we rejected the use of any vehicles which were used in metaphors that were rated less than 2 on a 1–6 scale in previous norming studies wherein we randomly paired topics with words varying on BOI.^1^ Lastly, each list of vehicles only had two animate items, which ought to further reduce unwanted variability that can occur with studying BOI, as low-BOI items tend to be animate while high-BOI items tend to be inanimate (Heard et al., 2019). The result was four conditions of vehicles, each with 12 items (i.e., high BOI-high SND; high BOI-low SND; low BOI-high SND; low BOI-low SND). See Table 2 for the full set of vehicle words.

**Table 2:** Words serving as potential vehicles and their respective semantic conditions.

High-BOI High-SND	High-BOI Low-SND	Low-BOI High-SND	Low-BOI Low-SND
Typewriter	Shovel	Butterfly	Lighthouse
Flashlight	Umbrella	Submarine	Volcano
Violin	Balloon	Statue	Pillar
Pillow	Wheelchair	Medal	Tiger
Ant	Pencil	Anchor	Pendulum
Bicycle	Puzzle	Airplane	Rainbow
Cigarette	Rat	Cannon	Palace
Seed	Clay	Dinosaur	Eagle
Wine	Hammer	Rocket	Lightning
Sword	Vacuum	Castle	Prairie
Camera	Gate	Storm	Cloud
Fish	Cat	Mountain	Desert

One-way ANOVAs were conducted to ensure the four vehicle conditions only differed on BOI and SND. The vehicles did not differ in concreteness, (*p* = 0.119); or frequency, (*p* = 0.952). Critically, the manipulation of BOI is significant, (*p* < 0.001), with Tukey-HSD tests confirming that the high-BOI items, across SND, do not significantly differ (*p* = 0.97) nor do the low-BOI items, across SND (*p* = 0.75), but the high and low BOI items differ from one another (both *p*’s < 0.001). The same can be said for the SND manipulation (*p* < 0.001), with Tukey-HSD tests confirming that high-SND items do not significantly differ (*p* = 0.99) nor the low-SND items (*p* = 0.90), but the high and low-SND items do (both *p*’s < 0.001).

### Procedure

Participants were instructed that their task was to create metaphors by choosing a vehicle which, with the presented topic, creates a comprehensible and apt metaphor. The instructions included *time is a river* and *time is a pickle* to demonstrate that not every topic-vehicle pairing is suitable. A practice trial involved the word *Time* along with the 48 vehicles. Participants were asked to choose a vehicle for the topic, and write out the entire metaphor on a provided sheet of paper. After this practice trial, the experimental trials followed. An experimental trial consisted of the presentation of a slide that included an abstract topic-word along with 48 vehicles presented below it. The order of the vehicles was pseudo-random such that each of the 36 slides contained a unique order of the vehicles. For each trial, participants were asked to choose a vehicle that, when paired with the presented topic, results in an apt and comprehensible metaphor, and subsequently wrote out the entire metaphor in the A is B format on their provided sheet in a numbered order from 1–36. In a subsequent task, participants were asked to interpret their metaphors, but this data is not included here as it was for a separate research question.

## Results

**Figure 1:**
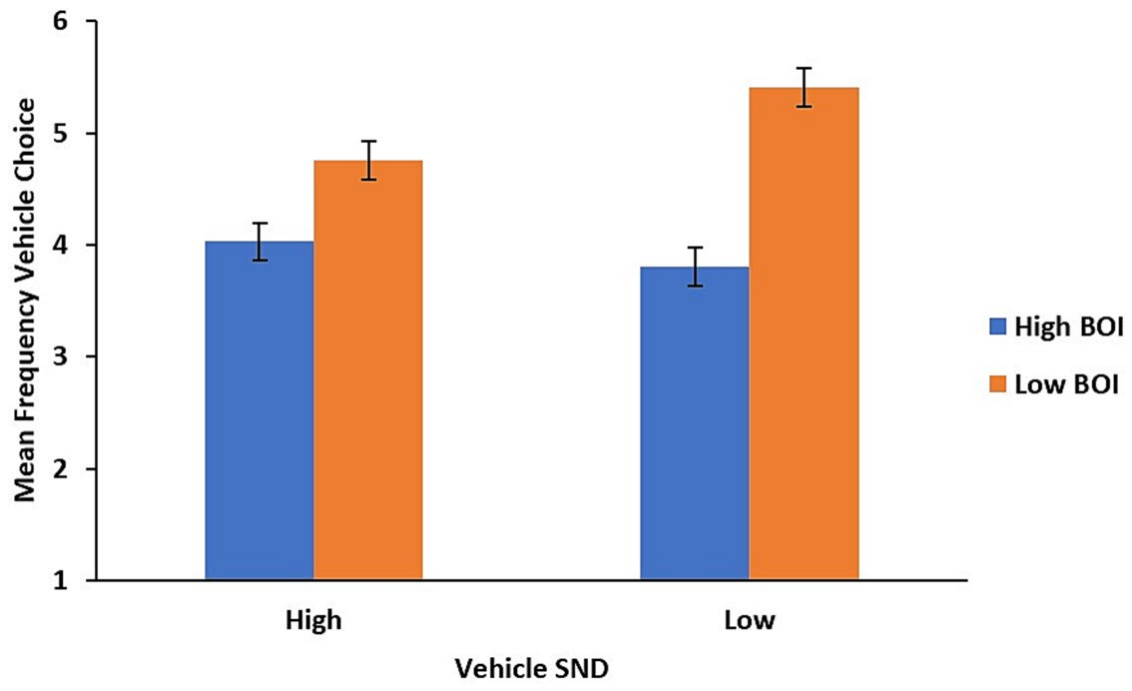
Vehicle SND by vehicle BOI interaction. Error bars represent standard error of the mean.

The data from 11 participants were not included in the analysis, for one or more of the following reasons: they did not complete the study, created many similes despite the instructions given, or created extended metaphors. Thus, the analyses are based on 49 participants. The resulting participants created and interpreted nominal metaphors of the form A is B (although 5 of which each created a single simile – we included such participants because a single simile may not necessarily be strategic, but possibly accidental). A topic (high-SND vs. low-SND) by vehicle BOI (high vs. low) by vehicle SND (high vs. low) repeated measures ANOVA was conducted on the frequency of vehicles chosen from the four semantic conditions. A main effect of BOI was obtained, *F* (1, 48) = 31.297, *p* = <0.001, η_p_^2^ = 0.395; participants chose low-BOI vehicles (*M* = 5.1, *SE* = 0.104) 56.4% of the time whereas they chose high-BOI vehicles (*M* = 3.9, *SE* = 0.103) less frequently at 43.5% of the time; however, this main-effect was qualified by two interactions. First, a vehicle-BOI by vehicle-SND interaction was observed, *F* (1, 48) = 9.883, *p* = 0.003, η_p_^2^ = 0.171. As is depicted in Figure 1, the effect of BOI differs for high and low-SND vehicles: low-BOI vehicles were preferred for both high and low-SND items but this preference was greater for low-SND vehicles than high-SND vehicles.

**Figure 2:**
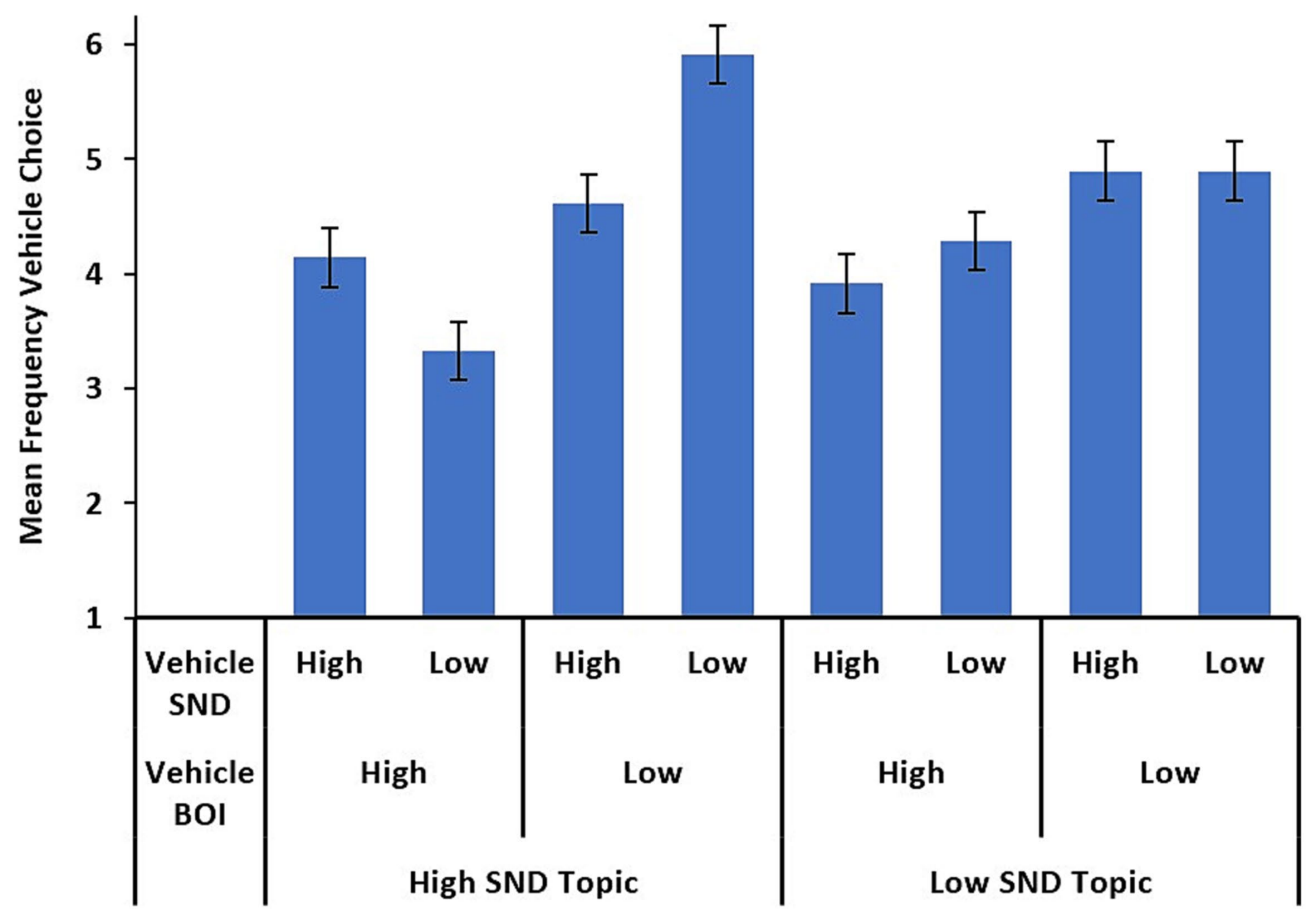
Condition means across levels of topic SND and vehicle SND and BOI. Error bars represent standard error.

Most importantly, a three-way, topic SND by vehicle BOI by vehicle SND, interaction was observed, *F* (1, 48) = 9.1, *p* = 0.004, η_p_^2^ = 0.159. As Figure 2 shows, the distribution of vehicle choices differs between high and low-SND topics. A simple-main effects analysis with vehicle BOI as the factor and topic and vehicle SND as the moderating factors revealed that, the low-BOI effect was present when the topic was high-SND and the vehicle was low-SND, *F* (1) = 40.74, *p* < 0.001 but not when the topic and vehicle were both high-SND, *F* (1) = 1.59, *p* = 0.21. Thus, for high-SND topics, participants relied more on low BOI – low SND vehicles to construct apt metaphors. We interpret this as an attempt by participants to reduce the overall semantic richness of the metaphor because the topic is already from a dense semantic neighborhood. Moreover, simple main effects analyses also revealed that when the topic was low-SND, the low-BOI effect was observed when participants selecting high-SND vehicles, *F* (1) = 8.27, *p* = 0.006, but no BOI effect was observed when both topic and vehicles were low-SND, *F* (1) = 2.17, *p* = 0.15. We interpret this to mean that when the topic is low SND, participants are drawn to low-SND vehicles, and higher semantic richness from BOI is tolerated. Moreover, when selecting a high-SND vehicle for a low-SND topic, participants preferred low-BOI words, again to reduce the richness from high-SND vehicles. Thus, when the topic is from a sparse semantic neighborhood, vehicle semantic richness does not matter as much as when the topic is from a dense semantic space.

In sum, participants did not choose the semantically richest vehicles when creating novel metaphors. Rather, our findings demonstrate that semantic richness is detrimental to metaphor processing, and that, on average participants aim to reduce semantic richness of the metaphor when selecting an apt metaphor vehicle to create a metaphor.

## Discussion

While Katz (1989) showed that concrete words serve as better metaphor vehicles than abstract words, our results characterize the nature of vehicle concreteness further. In general, our results showed that semantically less-rich concrete concepts are more amenable for metaphorical abstraction. In particular, we observed that sensorimotor and linguistic richness variables interact in novel metaphor production, such that participants reduced the overall semantic richness of their novel metaphors. This finding observed in the metaphor production task is consistent with recent work demonstrating that semantic richness is detrimental to novel metaphor comprehension. For example, Al-Azary and Buchanan (2017) constructed various novel metaphors varying on topic-concreteness and semantic neighborhood density of the topic and vehicle words, resulting in four conditions varying in semantic richness (abstract – low SND; concrete – low SND; abstract – high SND; concrete – high SND). They found that concreteness and semantic neighborhood density interacted, with semantically less-rich metaphors (low SND metaphors) being the most comprehensible, followed by abstract – high SND metaphors, and concrete – high SND metaphors, the semantically richest condition, being least comprehensible. To interpret their results, they argued that semantic richness, coming from topic concreteness and semantic neighborhood density, is detrimental to constructing metaphorical meaning, as such rich semantic representations are too specific and less malleable to take-on novel metaphoric associations. Moreover, irrelevant semantic properties, of which semantically richer concepts will have more of, must be inhibited during processing (Kintsch, 2000). Subsequent studied have reported similar findings in both offline and online tasks, and in special populations (Al-Azary et al., 2019, 2021). The present study extends findings of semantic richness to body-object interaction, and to metaphor production (rather than sole comprehension). We interpret the current results in a similar fashion to Al-Azary and Buchanan (2017); namely, semantically rich representations are too specified to take-on novel metaphorical meanings during metaphor processing. This was apparent here with the three-way interaction, as participants chose vehicles that were both low-BOI and low-SND when creating metaphors for high-SND topics.

The finding that low-BOI words served as better metaphorical vehicles on average (and especially when topic and vehicle words were high-SND) may be interpreted as evidence against embodied cognition, as such words are less amenable to motoric simulations than high-BOI counterparts. However, one must be cautious in such an interpretation, as the low-BOI words used in our study are nonetheless concrete concepts, and as such, can evoke sensory imagery. For example, low-BOI concepts such as *lighting* or *tiger* can trigger embodied simulations wherein one creates scenarios involving such concepts, even though one may not directly motorically interact with those words’ referents. Moreover, experimental work has demonstrated that bodily-actions related to concrete concepts are activated during metaphor processing (Al-Azary and Katz, 2021). Therefore, our results do not adjudicate between theoretical positions that necessitate a role for sensorimotor simulations in nominal metaphor comprehension (e.g., Gibbs and Matlock, 2008) or those that eschew sensorimotor simulations in favor of abstract representations (e.g., Glucksberg, 2008). However, our findings of low-BOI effects suggest that first-person motoric interaction, which BOI characterizes, is not the primary factor driving concreteness effects in metaphor production.

The semantic richness variables we considered – body-object interaction and semantic neighborhood density – characterize distinct sources of experience. Body-object interaction is learned through embodied experience whereas semantic neighborhood density reflects disembodied linguistic experience. The fact these two distinct variables interact in metaphor processing lends support to theoretical views that posit the presence of embodied and disembodied semantic representations that conjointly influence cognition (Paivio, 1971; Barsalou et al., 2008; Dove, 2011). Moreover, it has been argued that metaphor is a tool for building abstract representations from concrete concepts (Jamrozik et al., 2016). According to this “sensorimotor shedding” framework, novel metaphors are thought to be processed by sensorimotor simulations, and after repeated use, the sensorimotor properties begin to “shed,” making way for more abstract meanings to acrue. For example, consider *lemon*, a conventional word that metaphorically refers to something of little value due to its faultiness (e.g., *my car is a lemon*). Initially, *lemon* would be processed concretely, evoking multimodal simulations of taste, smell and feel, and after repeated exposure in linguistic contexts, develops an abstraction that is the primary representation when used metaphorically (see Al-Azary and Katz, 2021 for experimental support for this hypothesis). We speculate that low-BOI concepts, having less motor richness, have a “head start” in shedding their sensorimotor properties, and for this reason, are more amenable for metaphorical abstraction, as demonstrated in our study.

Other work has demonstrated that metaphorical potential is captured in a word’s relationality (Jamrozik et al., 2013). Accordingly, words such as *marriage* are more metaphorical than entity words, such as *knife*. Our work, however, demonstrates that among entities, there is considerable variation in metaphorical potential, which is predicted by word-level semantic representations such as semantic neighborhood density and body-object interaction. However, future work ought to characterize further the nature of metaphoricity in concrete concepts. For example, although concrete concepts are often defined in relation to imagery, imageability and concreteness are related yet distinct dimensions (Paivio et al., 1968). That is, some words are imageable, yet low on concreteness (such as *ghost*). Moreover, our focus was on motoric interaction, but other modalities in concrete concepts may play a role in metaphor, such as concepts with salient auditory (e.g., *thunder*) or motion (e.g., *bullet*) properties (see Cardillo et al., 2017 for modality specific metaphors). Furthermore, other measures of semantic richness have yet to be explored in metaphor processing experiments, such as the number of semantic features (McRae et al., 2005; Buchanan et al., 2019). Some concrete concepts are semantically rich because they have a high number of features, such as *toad* whereas others, like *guppy*, have fewer features and are semantically less-rich. More broadly, however, we acknowledge that pragmatic context can, and often does, make virtually any concept metaphorical, and can also shape a word’s intended metaphorical meaning (Gibbs et al., 2011; Haught, 2013). It should be the goal of future research to investigate the boundary conditions of semantic effects on metaphor processing, and how semantic representations interact with pragmatic contexts.

## Data availability statement

The raw data supporting the conclusions of this article will be made available by the authors, without undue reservation.

## Ethics statement

The studies involving humans were approved by the University of Western Ontario Research Ethics Board. The studies were conducted in accordance with the local legislation and institutional requirements. The participants provided their written informed consent to participate in this study.

## Author contributions

HA-A conceived and designed the experiment, collected and analyzed the data, and wrote the first draft of the manuscript. ANK played a supervisory role in every step of the process and supported the project with grant funding. All authors contributed to the article and approved the submitted version.

## Funding

This research was supported by Natural Sciences and Engineering Research Council of Canada Operating Grant NSERC 06P007040 to ANK.

## Conflict of interest

The authors declare that the research was conducted in the absence of any commercial or financial relationships that could be construed as a potential conflict of interest.

## Publisher’s note

All claims expressed in this article are solely those of the authors and do not necessarily represent those of their affiliated organizations, or those of the publisher, the editors and the reviewers. Any product that may be evaluated in this article, or claim that may be made by its manufacturer, is not guaranteed or endorsed by the publisher.

## Footnotes

^1^For example, in initial norming studies, the word *pelican* did not work in *Love is a pelican*, *Time is a pelican*, *Life is a pelican*, or *Diplomacy is a pelican*. It is likely that *pelican* may be an acceptable vehicle for a particular topic in an appropriate context, but we exclude its use for the current study. Moreover, vehicles in the BOI databases with somewhat similar meanings were reduced (e.g., *lion* and *tiger*) by choosing the one which satisfied other psycholinguistic constraints imposed on the stimuli (e.g., keeping frequency and concreteness matched). See Al-Azary (2018) Studies 1 and 2.
